# Knowledge and attitudes of parents and professionals to neonatal BCG vaccination in light of recent UK policy changes: A questionnaire study

**DOI:** 10.1186/1471-2334-7-82

**Published:** 2007-07-24

**Authors:** Morris Gordon, Hannah Roberts, Egware Odeka

**Affiliations:** 1 Department of Paediatrics, Royal Oldham Hospital, Oldham, UK

## Abstract

**Background:**

Universal BCG vaccination in the UK ended in 2005. The new vaccination policy instead offers a number of different forms of selective vaccination to newborns based on risk of acquiring TB. We set out to assess the attitudes and knowledge of both parents and professionals to the new policy for neonatal BCG vaccination.

**Methods:**

A short questionnaire was designed, made up of demographic and attitude questions, as well as very basic knowledge questions. The researchers handed out the questionnaire to all parents and professionals in the antenatal and postnatal areas, as well as the paediatric and neonatal units during a 6-week period. The site was the Royal Oldham hospital, a district general hospital with 3250 deliveries per year and multi-ethnic in its population mix.

**Results:**

A total of 253 completed questionnaires were collected. The ethnic origin of responders was 50.6% White British, 18.2% Bangladeshi, 8.7% Indian, 4% White/Asian, the remaining 18.5% of other origins. 71.5% of responders said they had heard of BCG vaccine. When asked if they knew the new policy for its use, 33.2% answered yes. 24.5% gave the most accurate response when asked who now receives BCG.

**Conclusion:**

We have found that amongst parents and professionals alike there is a lack of knowledge of the new policy. This has lead to confusion and as knowledge amongst the professionals who identify neonates for vaccination is low, uptake may be sub-optimal. We suggest that units investigate the issue and ensure that the new policy is understood and implemented correctly.

## Background

The UK BCG vaccination strategy for the last 50 years was based on universal vaccination of teenage school children if they had not previously been vaccinated, offering protection to the young adults in whom TB was most prevalent and most likely to be infectious [1,2]. In more recent years, a policy of selective vaccination was also introduced [3], providing protection to new immigrants and newborns perceived to be at high risk. Indeed, the BCG vaccine for newborns and infants has been shown to offer significant protection against TB [4]

Based on the changing make up of the UK population and the declining rates of TB in the age group in whom universal vaccination was taking place, the joint committee on vaccination and immunisation introduced new guidelines in July 2005, which were then made policy by the department of health [5]. The new policy [6] abolishes universal vaccination and instead offers vaccination to all newborns in areas of high TB incidence and selective vaccination in other areas, based on the country of origin of parents and grandparents [7].

We set out to assess the attitudes and knowledge of both parents and professionals to the new policy for the use of the BCG vaccine at the Royal Oldham hospital, a district general hospital with 3250 deliveries per year and multi-ethnic in its population mix.

## Methods

A short questionnaire was designed [see Additional file 1], made up of demographic and attitude questions, as well as very basic knowledge questions. This was piloted on a random sample of parents and professionals to check the clarity of the questions and appropriate language changes were made to allow the questions to gather the information required. The researchers handed out the questionnaire to all parents and professionals in the antenatal and postnatal areas, as well as the paediatric and neonatal units during a 6-week period. Health care workers who do not work in antenatal, postnatal or neonatal units were not asked to complete the questionnaire. A brief description of the study and explanation that participation was optional accompanied the questionnaire, no other guidance being offered by the interviewers. If a question was not understood, participants were asked to leave it blank. Patients who did not speak English were offered the use of the resident interpreters to complete the questionnaire. The completed questionnaires were collected immediately and data was coded and entered into SPSS for Windows version 11.5 for descriptive analysis (SPSS Inc, Chicago, USA).

## Results

A total of 253 completed questionnaires were collected. No one declined to take part in the study. Responders were made up of parents and professionals, consisting of 133 parents (52.6%), 63 midwifes (24.9%), 26 nurses (10.3%), 17 allied professionals (6.7%) and 14 doctors (5.5%). The majority of responders had zero (32%), one (36%) or two (18.6%) children. The ethnic origin of responders was 50.6% White British, 18.2% Bangladeshi, 8.7% Indian, 4% White/Asian, the remaining 18.5% made up of 12 other origins, with no one declining to disclose their origin.

71.5% had heard of BCG and 48.6% said they were aware of rules governing who receives it. 63.3% of professionals and 6.0% of parents asked said they were aware of the new 2006 policy that now governs who receives the vaccine. Looking at parents alone, 0.0% of those who had no children and 8.1% of those with children said they were aware of the new policy. Table 1 shows a summary of who responders thought receive BCG in this current policy. When broken down, 50.0% of professionals and 0.0% of parents asked chose the most accurate answer.

**Table 1:** Responses when participants were asked who currently receives BCG vaccine

Response	Frequency	Percentage
Don't Know	166	65.6
All babies	13	5.1
Some babies	62	24.5
All teenagers	8	3.2
Some teenagers	2	0.8
Only new immigrants	2	0.8

It is worth noting that this question is limited in its scope as it does not allow a responder to give a detailed response if they are fully aware of the new policy. However, after piloting the questions, it was found this was the best way of ascertaining whether responders were aware that selective neonatal vaccination is the mainstay of the new policy and vaccination in other age groups is reserved for immigrants or as catch up for those missed.

40 responders had looked for further information on the topic. Only 14 of the 40 said this information was useful. Finally, participants were asked to make any comments they wished. A summary of the most common responses is shown in Table 2.

**Table 2:** General comments recorded by participants

Comment	Frequency
Would like more information	26
Appears to be a racist policy at present	15
Have tried to find out information, but not been successful	7
Know about BCG, but not current policy	6
Doesn't care	6
People around me seem very confused	5
I don't know much about it	5
Thinks all babies should be getting it	4
Policy seems correct, but implementation is not	4
Has no knowledge and concerned as has other children who might need the BCG	2
Not enough leaflets for parents	2
Should be given by trained staff in a postnatal outpatient setting	2
There are too many vaccines	2
More emphasis needed on choice	2
Confused BCG with Vitamin K	2

## Discussion

The recent consultation by the national institute for clinical excellence [8] concluded that the school program was no longer cost effective in light of declining rates of TB in teenagers. The new policy for selective immunisation, shown in Table 3, offers targeted protection to newborns based on the rates of TB in their area of the UK [9] or their country of origin, as shown in Table 4.

**Table 3:** Newborn groups to be offered BCG vaccination [13]

• All babies living in areas where the incidence of TB is 40/100,000 or greater
• Babies whose parents or grandparents have lived in a country with a TB prevalence of 40/100,000 or higher
• Unvaccinated infant immigrants from countries with a high TB prevalence

**Table 4:** Countries with TB incidence greater than 40 per 100,000 in 2004 [14]

Afghanistan	Chad	Georgia	Malaysia	Peru	Thailand
Algeria	China	Ghana	Maldives	Philippines	Timor-Leste
Angola	China, Hong Kong	Guam	Mali	Portugal	Togo
Argentina	China, Macao SAR	Guatemala	Marshall Islands	Qatar	Turkmenistan
Armenia	Colombia	Guinea	Mauritania	Rep. Korea	Uganda
Azerbaijan	Comoros	Guinea-Bissau	Mauritius	Republic of Moldova	Ukraine
Bahrain	Congo	Guyana	Micronesia	Romania	UR Tanzania
Bangladesh	Côte d'Ivoire	Haiti	Mongolia	Russian Federation	Uzbekistan
Belarus	Croatia	Honduras	Morocco	Rwanda	Vanuatu
Belize	Djibouti	India	Mozambique	Sao Tome & Principe	Venezuela
Benin	Dominican Republic	Indonesia	Myanmar	Saudi Arabia	Viet Nam
Bhutan	DPR Korea	Iraq	Namibia	Senegal	Yemen
Bolivia	DR Congo	Kazakhstan	Nepal	Sierra Leone	Zambia
Bosnia & Herzegovina		Kenya	New Caledonia	Singapore	Zimbabwe
Botswana	Ecuador	Kiribati	Nicaragua	Solomon Islands	
Brazil	El Salvador	Kyrgyzstan	Niger	Somalia	
Brunei Darussalam	Equatorial Guinea	Lao PDR	Nigeria	South Africa	
Burkina Faso	Eritrea	Latvia	Northern Mariana Is	Sri Lanka	
Burundi	Estonia	Lesotho	Pakistan	Sudan	
Cambodia	Ethiopia	Liberia	Palau	Suriname	
Cameroon	Gabon	Lithuania	Panama	Swaziland	
Cape Verde	Gambia	Madagascar	Papua New Guinea	Syrian Arab Republic	
Central African Republic	Georgia	Malawi	Paraguay	Tajikistan	

Our hospital is in an area with less than 40/100,000 cases of TB and therefore as per the policy shown in Table 3, only new immigrant infants or infants whose parents or grandparents are from high risk areas should be offered BCG.

We have found that awareness of this policy is generally quite limited, with one third of responders not knowing what BCG is and two thirds not knowing the current policy for vaccination. It should be noted that lack of awareness of the new policy was widespread amongst professionals as well as parents. In particular only 27% of midwives were aware of the new policy and they are currently responsible for identifying those who need the BCG and informing the paediatricians who administer it. This was reflected in the comments made, with many midwives stating that they need more information and knowledge to inform parents effectively.

This general lack of knowledge seems to be having an impact on parents, with a number perceiving and then commenting that they felt the policy was racist. This is an understandable viewpoint with a policy that apparently vaccinates ethnic minorities with no clear explanation as to why and limited knowledge amongst professionals responsible for providing the relevant information. Wherever in the UK knowledge amongst professionals is limited, similar problems in the perceptions of the vaccine by parents may be seen. Also, it is also clear that when parents are motivated to find information they are generally unsatisfied (65% did not find information useful) and this may again be due to the lack of knowledge amongst professionals advising them.

We have discussed this with the local primary care trust (PCT) and they are working with the new policy within the area. They have educated all Health Visitors so that they can identify new immigrant infants who are eligible. In addition, all head teachers have been contacted and school age children have been sent a questionnaire to identify if they are eligible under the new policy. The immunisation coordinator has informed us that this has been well received and led to identification of many eligible children, as well as allowing concerns to be addressed. Unfortunately, our study has shown that this education program has not been introduced into secondary care in the area and both parents and professionals in this sector lack the knowledge needed to implement the new policy effectively for neonates. In trying to improve this situation, a general program of education surrounding the new policy and its implementation will be required for parents antenatally and for all professionals involved in their antenatal and postnatal care.

It has been previously suggested that vaccinating with BCG within the community in specialist clinics has a role [10]. This offers the advantage of being cost effective by using entire vials of vaccine. It also allows the vaccine to be given by someone very experienced both technically and in terms of their knowledge and could have a positive effect on understanding and awareness amongst parents. It has previously been suggested that vaccinating at birth is less effective than at three months [11], another reason to consider community clinics as an attractive alternative which needs further study.

We have shown in this study that lack of clarity as to the reasons for selective use of BCG causes anxiety amongst parents and it has been previously identified that white British infants who are eligible may not be vaccinated in such an environment [12]. Therefore, instigating a program aimed at identifying antenatally those eligible for BCG should increase uptake and educate all parents about the current policy and the reasons behind it. This well help allay concerns caused by the incorrect notion of a 'racial' factor in the use of neonatal BCG, as commented on by several respondents in this study (Table 2).

## Conclusion

We have found that amongst parents and professionals alike there is a significant lack of knowledge of the new BCG administration policy. In our district general hospital, this has lead to much confusion and as knowledge amongst the professionals who identify neonates for vaccination is low, uptake may be sub-optimal. We suggest that units investigate the issue and ensure that the new policy is understood and implemented correctly. If problems are being encountered, a clear policy of antenatal education of parents and identification of eligible babies will ensure an appropriate uptake of BCG, as well as addressing concerns as to the distribution of the vaccine by the new policy by improving knowledge and understanding.

## Abbreviations

BCG – Bacillus Calmette-Guerin

TB – Tuberculosis

## Competing interests

The author(s) declare that they have no competing interests.

## Authors' contributions

MG participated in the design of the study, its coordination and drafted the manuscript. HR participated in the design and collected the data. EO conceived of the study, and participated in its design and coordination. All authors read and approved the final manuscript.

## Pre-publication history

The pre-publication history for this paper can be accessed here:



## Supplementary Material

Additional file 1 Questionnaire. The questionnaire as used for this study. Click here for file
