# Supplementary material for: Knowledge and attitudes of parents and professionals to neonatal BCG vaccination in light of recent UK policy changes: A questionnaire study
Source: BMC Infect Dis. 2007 Jul 24;7:82. doi: 10.1186/1471-2334-7-82 (PMC1940004; doi:10.1186/1471-2334-7-82)
Supplement: Additional file 1 — Questionnaire. The questionnaire as used for this study. [file 1471-2334-7-82-S1.doc]

# **Additional file 1.**

# **Questionnaire**

1) Are you here today as a: - Parent / Doctor / Nurse / Midwife / Allied professional

2) How many children do you already have? ____________

3) I would describe my ethnic origin as follows:-

# Asian or Asian British

Bangladeshi

Indian

Pakistani

Any other Asian background

# Mixed

White & Asian

White & Black African

White & Black Caribbean

Any other mixed background

Any other ethnic group

African

# Other Ethnic Group

# Chinese

# Black or Black British

Caribbean

Any other Black background

# White

British

Irish

Any other White background

I do not wish to disclose my

ethnic origin

4) Are you aware there is a vaccine called BCG used for TB? (SHOW LEAFLET) Yes/No

5) Are you aware of any rules governing who receives the BCG vaccine? Yes/No

6a) Do you know of the new policy (2006) for the use of BCG vaccine? Yes/No

6b) **If yes** which one choice best describes who is offered the vaccine in the new policy?

All babies / some babies / all teenagers /some teenagers /

only new immigrants and no one else / other

6c) **If yes** do you think the policy of who gets it is correct? Yes/No

7a) Have you ever tried to find out more about the BCG vaccine? Yes/No

7b) **If yes** where did you find your information?

GP/Hospital/Books/Internet/Friends or family/Information Leaflet/TV/Radio/other

7c) If you found information, was it useful? Yes/No

COMMENTS_________________________________________________________
